# The Cumulative Risk of Prenatal Exposures to Chemical and Non-Chemical Stressors on Birth Outcomes in Suriname

**DOI:** 10.3390/ijerph18147683

**Published:** 2021-07-20

**Authors:** Anisma R. Gokoel, Arti Shankar, Firoz Abdoel Wahid, Ashna D. Hindori-Mohangoo, Hannah H. Covert, Jeffrey K. Wickliffe, Emily W. Harville, Wilco C. W. R. Zijlmans, Maureen Y. Lichtveld

**Affiliations:** 1 Scientific Research Center Suriname, Academic Hospital Paramaribo, Paramaribo, Suriname; 2 Faculty of Medical Sciences, Anton de Kom University of Suriname, Paramaribo, Suriname; wilco.zijlmans@uvs.edu; 3 Department of Biostatistics and Data Science, School of Public Health and Tropical Medicine, Tulane University, New Orleans, LA 70112, USA; sarti@tulane.edu; 4 Graduate School of Public Health, University of Pittsburgh, Pittsburgh, PA 15261, USA; fabdoel72@gmail.com (F.A.W.); mlichtve@pitt.edu (M.Y.L.); 5 Department of Environmental Health Sciences, School of Public Health and Tropical Medicine, Tulane University, New Orleans, LA 70112, USA; ashna.mohangoo@perisur.org (A.D.H.-M.); hcovert@tulane.edu (H.H.C.); 6 Foundation for Perinatal Interventions and Research in Suriname (Perisur), Paramaribo, Suriname; 7 School of Public Health, University of Alabama at Birmingham, Birmingham, AL 35294, USA; jkwickli@uab.edu; 8 Department of Epidemiology, School of Public Health and Tropical Medicine, Tulane University, New Orleans, LA 70112, USA; eharvill@tulane.edu

**Keywords:** chemical stressors, non-chemical stressors, birth outcomes, cumulative exposure, path model, Suriname, CCREOH–MeKiTamara study

## Abstract

The cumulative exposure to chemical and non-chemical stressors may have an impact on birth outcomes. The aim of this study is to examine the cumulative exposure of a mixture of chemicals (mercury, lead, selenium and tin) and non-chemical stressors (social support, perceived stress, probable depression and BMI) on birth outcomes (birthweight, gestational age at birth, and Apgar score at 5 min). The study population is a subset (*n* = 384) of the Caribbean Consortium for Research in Environmental and Occupational Health–MeKiTamara prospective cohort study. Associations between the latent chemical construct, non-chemical stressors and birth outcomes were assessed using path models. The results showed a significant direct relationship between perceived stress and birthweight (β = −0.17), however even though the relationship between perceived stress and depression was significant in all three path models (β = 0.61), the association between depression and birth outcomes was not significant. Perceived stress was significantly associated with community engagement (β = −0.12) and individual resilience (β = −0.12). BMI (β = 0.12) was also significantly directly associated with birthweight. The latent chemical construct did not show an association with the birth outcomes. Our data indicate the need for the development of a support system for pregnant women by involving them in prenatal care programs to reduce maternal stress, which may also influence depression and (in)directly improve the birth outcomes. Interventions regarding weight management for women of childbearing age are necessary to halt obesity and its negative effects on birth outcomes.

## 1. Introduction

Chemical and non-chemical stressors have been found to be independently associated with adverse birth outcomes such as low birthweight (LBW), low gestational age at birth and low Apgar score [1,2,3,4,5]. High levels of mercury (Hg) in maternal blood and erythrocytes are significantly associated with decreased birthweight [2,3]. In addition, high levels of selenium (Se) in pregnant women have been linked with congenital heart defects (CHDs) [6]. However, evidence also suggests that Se, which is an essential nutrient, has a mitigating effect and can counteract the toxicity of Hg [7,8,9]. Maternal prenatal blood lead (Pb) levels ≥10 ug/dL have proven to pose a high risk for preterm birth (PTB) or small-for-gestational-age (SGA) babies [10]. Non-chemical stressors including stress, depression and low social support may also negatively influence birth outcomes, such as gestational age at labor, birthweight and neurodevelopmental outcomes [5,11,12,13,14].

While there is evidence of an association between either some non-chemical stressors or chemicals and birth outcomes, only a few studies have been conducted that study the cumulative and combined exposure of non-chemical stressors and chemicals on birth outcomes [11,12]. In addition, the impact of some chemicals and elements such as tin (Sn) as well as mixtures on birth outcomes have not yet been well investigated [15]. To our knowledge, environmental health studies in the Caribbean, including Suriname, have not considered the complex mixture of chemicals and non-chemical stressors on birth outcomes.

Suriname, a middle-income country in South America, has a multi-ethnic population, consisting of people of Asian (41.1%; Hindustani and Javanese), African (38.1%; Tribal People and Creoles), Indigenous (3.8%), “Other” descent (16.4%; Mixed, Chinese, and Caucasians), and unknown (0.6%) [16]. The hospital-based late stillbirth rate (stillbirth later than 28 weeks of gestation, or birthweight ≥1000 g) is estimated at 16 per 1000 births (2016–2017). In 2019, Suriname had an overall neonatal mortality rate (NMR) of 11.2 per 1000 live births, higher than the NMR in Latin America and the Caribbean from 2008–2014 of 9 deaths per 1000 live births [17,18,19]. The prevalence in Suriname of PTB (<37 weeks of gestation) and LBW (<2500 g) is respectively 15% and 13% [18,20], higher than the LBW and PTB of the entire Latin America and the Caribbean region (respectively 13% and 9%) [21]. The rates of PTB and LBW in Suriname are in line with those in Jamaica and Guyana, but are higher than Cuba, Mexico and Venezuela [22]. Findings of the Caribbean Consortium for Research in Environmental and Occupational Health (CCREOH)–MeKiTamara study showed high maternal levels of hair Hg (≥1.1 µg/g) in 37.5% of participants, high perceived stress (score ≥ 20) in 27.2% and probable depression (score ≥ 12) in 22.4% [20].

Despite the potential exposure to high levels of Hg, perceived stress, and probable depression, and high rates of LBW and PTB in Suriname, to date no studies have examined the combined effect of the cumulative exposure of chemicals and non-chemical factors on birth outcomes in Suriname. The aim of this study is to examine the cumulative exposure of a mixture of chemicals (Hg, Pb, Se and Sn) and non-chemical stressors (social support, perceived stress, probable depression and BMI) on birth outcomes in Suriname.

## 2. Materials and Methods

### 2.1. Study Design and Setting

This study is part of the Caribbean Consortium for Research in Environmental and Occupational Health (CCREOH)–MeKiTamara study, which is a prospective environmental epidemiological cohort study. Pregnant women were recruited during the first or second trimester of pregnancy in three regions of Suriname: Paramaribo, Nickerie and the Amazonian Interior. The types and levels of exposures are expected to be different among these regions, with respect to both non-essential and essential elements, which may be explained by their diets and metal-based pesticides [23].

### 2.2. Study Population

The study population is a subset of the MeKiTamara study that recruited participants from December 2016 to July 2019 (*n* = 1189). The subset of 384 participants from the CCREOH cohort, is a statistically acceptable representation of the entire study cohort (*n* = 1190). Women were eligible if they were 16 years or older, spoke Dutch, Saramaccan, or Trio, had a singleton gestation, were planning to give birth at one of the study sites and provided written informed consent/assent. Pregnant women were recruited at four hospitals, prenatal clinics and midwife facilities of the Regional Health Department, and at multiple health care clinics of the Medical Mission Primary Health Care Suriname (MMPHCS) in the Interior [20,23].

### 2.3. Data Collection

Cohen’s Perceived Stress Scale (PSS), the Edinburgh Depression Scale (EDS), and the Social Support List-Interactions-12 (SSL-I-12), all self-report questionnaires, were administered by trained recruiters through face-to-face interviews during the first or second trimester of pregnancy using encrypted iPads. Data on height and weight were also collected at inclusion and used to calculate BMI. BMI calculations were based on body weight during pregnancy. Birth outcomes were collected after birth from parturition records. These data were uploaded in a Research Electronic Data Capture (REDCap) project site database for data cleaning and analysis purposes. REDCap is a secure web application for building and monitoring online surveys and databases to collect data for research purposes and can be used online or offline [20,24].

Maternal blood was collected at enrollment during either the first or second trimester. Whole blood was collected by venipuncture into 10 mL trace element vacutainers containing K2EDTA. Samples were kept cold (4 °C) for no more than 24 h prior to processing. Two mL aliquots of each sample were transferred to plastic cryovials and then stored at −80 °C.

### 2.4. Exposures and Covariates

Chemicals: Solubilization and analysis of the whole blood samples for the chemicals (Hg, Pb, Se, Sn, Manganese (Mn) and Cadmium (Cd)) by magnetic-sector ICPMS (SF-ICPMS) were performed by the Trace Element Research Group of the Wisconsin State Laboratory of Hygiene (WSLH) at the University of Wisconsin-Madison, United States. Sample preparation and analysis were carried out in a Class 1000 ISO 6 trace element clean lab at the WSLH. Critical sample handling steps were performed under HEPA-filtered (CLASS 100) all-polypropylene biosafety cabinets.

Perceived stress: This was assessed using the Cohen’s Perceived Stress Scale (PSS). The PSS is a widely used measure of perceived stress that has been validated in a number of populations and languages, including Dutch, which is the formal language in Suriname and, in line with our inclusion criteria, the questionnaire was thus administered in Dutch [25,26,27,28]. The questionnaire included ten items about the degree of experiencing stress due to having no control over things, nervousness, and not feeling confident in the past four weeks. The five response options were: 0 for never, 1 for almost never, 2 for sometimes, 3 for fairly often and 4 for very often. The total score ranges from 0 to 40 points; 0 indicates the lowest stress level and 40 the highest stress level [20].

Probable depression: This was assessed using the Edinburgh Postnatal Depression Scale. This questionnaire has also been validated for assessing prenatal depression. For prenatal purposes, the scale is called the Edinburgh Depression Scale (EDS) [29]. The EDS includes ten items concerning anxiety and depression symptoms on a four-point Likert scale: 0 = yes, very often; 1 = yes, mostly; 2 = no, not often; and 3 = no, not at all. A total depression sum score of all statements ranges from 0 to 30 points. A higher total depression score indicates a higher risk of probable depression.

Social support: Social support was assessed using the Social Support List-Interactions-12 (SSL-I-12), which includes twelve statements about support, affection, and attention from family and friends. There are four response options: 1 for rarely or never, 2 for occasionally, 3 for regularly and 4 for very often. Before data collection, one question (“Does it ever happen that people confide in you?”) was deleted, because of concerns regarding subjectivity based upon Suriname’s cultural context. Since the SSL-12-I scale was modified, an exploratory factor analysis was implemented, resulting in a two-factor solution: the Individual Resilience subscale (characteristics such as support and advice to allow individuals to adapt to adverse conditions) and the Community Engagement subscale (affection/attention from the community) [20].

Demographic Data: Demographic variables were categorized into the following groups: age at intake (16–19, 20–34, or ≥35 years), household income (<3000, or ≥3000 SRD (USD 143)), educational level (no, primary or lower secondary, upper secondary or tertiary); race/ethnicity: African descent (Creole, Tribal), Asian descent (Hindustani, Javanese), or other (Caucasian, Indigenous, Mixed)); BMI (continuous); and region (urban (Paramaribo, Wanica), rural (Commewijne, Saramacca, Para, Nickerie and Coronie) and the Interior (Marowijne, Brokopondo and Sipaliwini).

Birth Outcomes: Information on gestational age, birthweight and Apgar score was obtained from parturition books completed by midwives in the hospitals, prenatal clinics or midwife facilities where the baby was born.

### 2.5. Data Analysis

The distributions of chemicals were tested for normality using the Kolmogorov Smirnov test (*p* ≥ 0.05). The data were log transformed because the normality assumption was not met. Even after the log transformation the data were slightly skewed. To account for the skewness the ULS method was used for exploratory factor analysis (EFA). Measurements were done on Hg, Pb, Se, Sn, Mn and Cd. The data were randomly divided into the test and the validation sample. The EFA was conducted on the test sample to reduce the six chemicals into a smaller number of latent construct. The EFA resulted in a one factor solution with high factor loadings of Hg, Pb, Se, and Sn on this factor. Evaluation of the EFA models was based on eigenvalues, amount of variance explained, factor loadings higher than 0.35 and no cross loading greater than 0.25. We used confirmatory factor analysis (CFA) to confirm the latent constructs using the validation sample. The EFA resulted in a one factor solution with no cross loadings (Table 1).

Furthermore, the t-test was used for significance of the computed Pearson correlation coefficient. We then built a path model using structural equation modelling (SEM) to estimate the associations between the latent chemical construct and non-chemical stressors in predicting birthweight, gestational age and Apgar score. The path model was based on the fit criteria for the SEM models, which included several tests: the Chi-square (*p* > 0.05), Root Mean Square Error of Approximation (RMSEA) of 0.05, and the adjusted goodness-of-fit index (AGFI) with values greater than 0.95 indicating good fit (Bentler–Bonett Normed Fit Index (NFI ≥ 0.90), and Bentler’s Comparative Fit Index (CFI ≥ 0.90)). Lagrange Multiplier (LM) test was used to identify new causal paths if the fit criteria were not met. The best fit model included the following variables: the latent construct (Hg, Pb, Se and Sn), non-chemical stressors (social support, perceived stress, probable depression and BMI) and the birth outcomes (gestational age, birthweight and Apgar score). Covariates such as age, ethnicity, income, education and region were used to describe the sample, but were not part of the path model. The statistical package that was used to analyze the data is SAS 9.4.1 (SAS Institute, Cary, NC, USA).

### 2.6. Ethical Considerations

This study was approved by the Institutional Review Board (IRB) of Tulane University (number: 839093) and the Medical Ethical Commission of Suriname’s Ministry of Health (VG 023-14). All women 18 or older gave written informed consent, and assent was obtained from women who were 16 or 17 years old.

## 3. Results

The median levels of perceived stress, probable depression, community engagement and individual resilience were respectively 17.0 (IQR 13.0–20.0), 7.0 (IQR 4.0–11.0), 4.0 (IQR 4.0–6.0) and 25.0 (IQR 21.0–28.0). The median age of the participants was 28.2 years (IQR 24.2–32.7). The majority of the participants were of African descent (50.0%), followed by Asian descent (29.7%) and other/mixed ethnicities (20.1%). The majority of the participants had lower household incomes (59.1%) and were less educated (54.2%). The median BMI of the participants was 25.9 (IQR 22.6–30.8). The largest group (69.0%) of the participants resided in an urban area. Median concentrations of Hg, Pb, Se and Sn were 2.9 ug/L (IQR 1.7–4.6), 2.0 ug/dL (IQR 1.3–3.1), 191.2 ug/L (IQR 167.4–217.7) and 0.7 ug/L (IQR 0.5–1.0), respectively (Table 2).

The variables that were significantly correlated with each other were probable depression and perceived stress (r = 0.61, *p* < 0.001), gestational age and birthweight (r = 0.65, *p* < 0.001), gestational age and Apgar score (r = 0.55, *p* < 0.001), and birthweight and Apgar score (r = 0.46, *p* < 0.001). A negative correlation was found between perceived stress and individual resilience (r = −0.16, *p* < 0.001), community engagement (r = −0.16, *p* < 0.001) and birthweight (r = −0.14, *p* = 0.01). A correlation also was observed between community engagement and individual resilience (r = 0.34, *p* < 0.001), and between pregnancy-BMI and birthweight (r = 0.13, *p* = 0.03) (Table 3).

**Figure 1:**
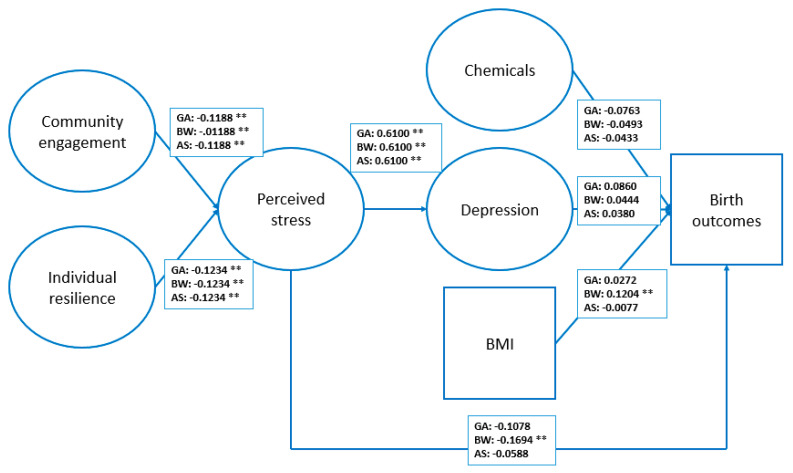
Path model of relationships among non-chemical stressors, the latent chemical construct, and birth outcomes. GA: gestational age model; BW: birthweight model; AS: Apgar score model. ** Significant at <0.05 level. Circle implies latent construct and square implies manifest variable.

To examine the cumulative exposure of the non-chemical stressors and the latent chemical construct, three path models were constructed. There was a significant direct relationship between perceived stress and birthweight (β = −0.17); however, even though the relationship between perceived stress and depression was significant in all three path models (β = 0.61), the association between depression and birth outcomes was not significant. Perceived stress was significantly associated with community engagement (β = −0.12) and individual resilience (β = −0.12) (see Figure 1). BMI (β = 0.12) was also significantly directly associated with birthweight.

Results of the structural equation path models (Table 4) showed good model fit of the final models. The gestational age model predicting the associations between the latent chemical construct, social support, perceived stress, probable depression and BMI on gestational age showed good model fit (χ^2^ = 11.00, Df = 8, *p* = 0.20, RMSEA = 0.03, CFI = 0.99 and NFI = 0.95) (Table 4). The model of birthweight yielded a good fit for the latent chemical construct, social support, perceived stress, depression and BMI (χ^2^ = 9.64, Df = 8 *p* = 0.29, RMSEA = 0.02, CFI = 0.99 and NFI = 0.96). The model predicting the associations between the latent chemical construct, social support, perceived stress, probable depression, BMI and Apgar score also resulted in good model fit (χ^2^ = 10.63, Df = 8, *p* = 0.22, RMSEA = 0.03, CFI = 0.99 and NFI = 0.95).

## 4. Discussion

Modeling exposures cumulatively using a path model may explain how different levels of chemicals together may affect birth outcomes [11]. The analysis of the path models indicated that multiple non-chemical stressors could have a cumulative effect on the distribution of gestational age at birth, birthweight and low Apgar score. Our study did not find a significant association between the combined chemical latent factor and any birth outcomes. These results are consistent with other studies examining the association of chemicals and non-chemical stressors on birth outcomes [11,20,30].

The lack of a statistically significant association between the combined chemical latent factor and birth outcomes may be explained by the concentrations of the chemical contaminants, which may be too low to find an association, or that the non-chemical factors outweighed any effects of the chemical exposure. The latter phenomenon was also observed in a study by Pao et al. (2019) in which socioeconomic variables played a dominant role and outweighed other effects, including environmental exposure [11]. Thomas et al. (2015) also found no association between blood Pb, Cd or arsenic and gestational age [30]. A review study by Vesterinen et al. (2017), however, found that smoking in combination with high stress significantly reduced BW. In addition, air-pollution exposure combined with high stress resulted in decreased BW [31]. Another reason for not finding an association between the combined chemical latent factor and birth outcomes may be the possibility of interactions among individual contaminants and Se as an essential element. In-depth analyses showed a significantly high correlation between Se and Hg (r = 0.61). Research has shown that Se-enriched diets may be protective against methylmercury toxicity [7,8]. Lastly, to our knowledge the combination of chemical exposure (Pb, Hg, Sn) and concentrations of Se as an essential element and non-chemical stressors (stress, probable depression, social support, BMI) that we included in our study is unique compared to previous studies of combinations of chemical exposure and non-chemical factors. To date, limited research has been conducted on the combined evaluation of exposure to environmental toxicants and psychosocial stressors [11,30,31,32].

Our results are also in line with our previous study of nearly the entire study population (n = 1143) where we did not find an interaction between depression, stress and Hg, but observed associations between socio-demographic factors, perceived stress, and birth outcomes [20]. The study described here builds on our previous research by combining several chemicals and non-chemical stressors using the path models to assess the cumulative exposure on birth outcomes.

The (in)direct association of social support with perceived stress, depression and birthweight is in line with previous studies [4,5]. A prospective study in Iran of 500 pregnant women showed that perceived social support directly through socioeconomic status and indirectly through anxiety, perceived stress, and probable depression affected gestational age at birth and birthweight [5,33]. In our study, social support indirectly affected the birthweight through perceived stress. We found a negative association between social support and perceived stress.

Perceived stress was directly significantly associated with birthweight. In the birthweight model, higher levels of perceived stress were associated with low birthweight. This was also seen in a study of 279 pregnant women in a suburban area in the United States, with some similarities to our study regarding demographic variables such as age and education. This U.S.-based study showed that latent pregnancy-specific stress could predict adverse birth outcomes better than other latent factors such as state of anxiety, perceived stress, life event stress and a combined latent factor constructed from all stress measures. Pregnancy-specific stress contributed directly to preterm birth and indirectly through its association with smoking to LBW [34]. There was a relatively strong association between perceived stress and probable depression, which would indicate that if stress during pregnancy increases, the risk of depression during pregnancy also increases. However, depression did not show a direct association with the birth outcomes, which may be explained by the relatively small sample size. A previous study of the CCREOH cohort examining the influence of perceived stress on depression showed a prevalence of perceived stress of 27.4% [35]. This high prevalence of perceived stress in combination with other exposures such as socioeconomic status and other maternal risk factors may explain the association with birthweight. Approximately 55% of our participants had no education, primary education or lower secondary/vocational education, and about 65% had monthly household incomes of lower than SRD 3000 (equivalent to USD 143). The sample of this study is a statistically acceptable representation of the total CCREOH cohort [23].

In the birth outcome model, the pregnancy BMI of the mother showed a positive direct association with birthweight. A systematic review and meta-analysis of the risk of maternal BMI and neonatal adverse outcomes in China revealed that high maternal BMI compared to normal maternal BMI was associated with fetal overgrowth, defined as macrosomia ≥4000 g, and increased risk of preterm birth [36,37,38,39]. Taking our sample into account, in depth analyses showed a significant association between maternal BMI and macrosomia (*p* = 0.042). Women in our study with a high maternal BMI (55.9% overweight or obese) compared to a normal or underweight BMI were over six times more likely to give birth to a macrosomic infant. A systematic review and meta-analysis found that mothers that were obese (as measured by BMI) prior to conception were more likely to give birth to a child with obesity with increasing child age [40]. Although our study took pregnancy BMI into account instead of maternal pre-pregnancy BMI, we assume that the pregnancy BMI of our sample size does not differ significantly from the pre-pregnancy BMI, since this was measured during early pregnancy. However, we did not take gestational age into account, because we believe that this has minimal impact on our study results since the independent and dependent variables such as metal concentrations, probable depression, perceived stress, social support and BMI did not differ among the different trimesters. Previous studies showed that maternal obesity could increase the risk of preterm birth, childhood overweight/obesity, autism spectrum disorder, offspring depression, anxiety, schizophrenia and eating disorders, so it could have long-term adverse health outcomes for the child [41,42,43].

### Limitations

There are some limitations to the study. The psychosocial questionnaires were not specifically validated for the Surinamese setting before data collection. However, these questionnaires are standardized and are widely utilized in research settings, including in low- and middle-income countries (LMICs) [14]. In addition, explanatory factor analysis was conducted before data analysis, which showed high factor loadings on the factor(s) and no cross loadings [35]. Probable depression was measured with a screening tool and not clinically assessed by a mental health specialist. Hence, the prevalence of probable depression may potentially differ from the actual prevalence of depression. However, this is a minor limitation since the EDS questionnaire does not produce artificially high scores [14]. We used pregnancy BMI only. While pre-pregnancy BMI is often preferred in studies since women gain weight during pregnancy [11,44], our participants were already pregnant at enrollment as defined by our inclusion criteria, and we did not collect self-reported pre-pregnancy weight due to concerns with recall. Pre-pregnancy weight is not routinely reported in primary care in Suriname, especially not in the interior, and hence not readily available in medical records. However, using pregnancy BMI only had minimal impact on our study results since BMI across participants did not differ significantly between trimesters, as was the case for all other independent and dependent variables. In addition, dietary habits, which may affect birth outcomes [45], were not accounted for in this study. However, it is likely that environmental exposures associated with the diet are at least partially reflected in the metal biomarker data [46,47]. For example, women who frequently consume predatory fish likely had higher Hg levels than those who do not [48]. In this study, some women were included during the second trimester even though it would have been ideal to recruit only in first trimester. In Suriname, and as reflected in our study, some women do not seek prenatal care until the second trimester for cultural and health-access reasons. While this is a limitation, the emphasis of our overall study is on environmental exposures and neurodevelopment in children, not only on birth outcomes. Therefore, conducting an exposure assessment through biomarker analysis remains of value to examine the relationship between prenatal environmental exposure and neurodevelopment in the child. As with many other studies, we were unable to measure or account for every factor that might influence birth outcomes, such as (chronic) diseases or socio-demographic factors.

## 5. Conclusions

This study is the first to examine cumulative exposure to environmental contaminants and concentrations of Se as an essential element and non-chemical stressors in Suriname and the entire Caribbean region. Results indicate that combined exposures may influence birth outcomes, with the influence of non-chemical stressors being of particular importance. Our data indicate the need for the development of a support system for pregnant women by involving them in prenatal care programs to reduce maternal stress, which may also influence depression and (in)directly birth outcomes. Interventions regarding weight management for women of childbearing age are necessary to halt obesity and its negative effects on birth outcomes. We suggest further research on the cumulative exposure of chemicals and non-chemical factors, including a larger sample size, more socio-demographic risk factors, and a better assessment of any history of diseases of maternal health and mental health.

## Figures and Tables

**Table 1:** Exploratory (n = 192) and confirmatory (n = 192) factor analysis.

EFA Results		CFA Results	
Factor Pattern	Factor Loadings	Fit Indices	
Mercury (ln)	0.77	Goodness of Fit Index (GFI)	0.98
Lead (ln) Selenium (ln) Tin (ln) Eigenvalue Variance explained	0.64 0.61 0.35 1.50 15.04%	Adjusted Goodness of Fit Index (AGFI) Parsimony Goodness of Fit Index	0.99 0.83

**Table 2:** Characteristics of the study population.

Characteristic	Total *n* (%)
Total	384 (100)
Non-chemical stressors	
Perceived stress (median, IQR)	17.0 (13.0–20.0)
Probable depression (median, IQR)	7.0 (4.0–11.0)
Community engagement (median, IQR)	4.0 (4.0–6.0)
Individual resilience (median, IQR)	25.0 (21.0–28.0)
Age (years)	
Median (IQR)	28.19 (24.2–32.7)
16–19	37 (9.6)
20–34	291 (75.8)
35+	56 (14.6)
Ethnicity (self-reported)	
African descent	192 (50.0)
Asian descent	114 (29.7)
Other/mixed	77 (20.1)
Missing	1 (0.2)
Household income (in SRD)	
<3000	227 (59.1)
≥3000	136 (35.4)
Missing	21 (5.5)
Educational Level	
None, primary, lower secondary/vocational	208 (54.2)
Upper secondary/vocational or tertiary	176 (45.8)
BMI	
Median (IQR)	25.9 (22.6–30.8)
Underweight (<18.5 kg/m^2^)	20 (5.2)
Normal (18.5–24.9 kg/m^2^)	133 (34.6)
Overweight (25–29.9 kg/m^2^)	90 (23.4)
Obese (≥30 kg/m^2^)	104 (27.1)
Missing	37 (9.6)
Region	
Urban	265 (69.0)
Rural	82 (21.4)
Interior	37 (9.6)
Missing	0 (0)
Concentrations of chemicals (median, IQR)	
Hg (ug/L)	2.9 (1.7–4.6)
Pb (ug/dL)	2.0 (1.3–3.1)
Se (ug/L)	191.2 (167.4–217.7)
Sn (ug/L)	0.7 (0.5–1.0)

**Table 3:** Pearson correlation coefficients of latent chemical construct, depression, perceived stress, social support, BMI and birth outcomes.

Prob > |r| under H0: Rho = 0	Se_Sn_Hg_Pb	Depression	Stress	Ind_res ^1^	Comm_eng ^2^	BMI	GA ^3^	BW ^4^	Apgar Score
Se_Sn_Hg_Pb *p*-value	1.00	0.04	−0.07	0.02	0.03	−0.02	−0.06	−0.04	−0.04
		0.47	0.19	0.67	0.54	0.66	0.24	0.47	0.49
Depression *p*-value		1.00	0.61 **	−0.10	−0.03	−0.06	0.02	−0.07	0.00
			<0.001	0.06	0.61	0.28	0.74	0.22	0.99
Stress *p*-value			1.00	−0.16 **	−0.16 **	−0.04	−0.05	−0.14 **	−0.03
				0.002	0.002	0.45	0.37	0.01	0.56
Ind_res ^1^ *p*-value				1.00	0.34 **	0.04	−0.02	0.00	0.01
					<0.001	0.44	0.71	0.97	0.86
Comm_eng ^2^ *p*-value					1.00	−0.01	0.07	0.05	0.08
						0.83	0.18	0.38	0.14
BMI *p*-value						1.00	−0.03	0.13 *	0.01
							0.65	0.03	0.87
GA ^3^ *p*-value							1.00	0.65 **	0.55 **
								<0.001	<0.001
BW ^4^ *p*-value								1.00	0.46 **
									<0.001
Apgar score *p*-value									1.00
								

* Correlation is significant at the 0.01 level (2-tailed). ** Correlation is significant at the 0.05 level (2-tailed). ^1^ Individual resilience, ^2^ Community engagement, ^3^ Gestational age, ^4^ Birthweight.

**Table 4:** Fit indices for models’ gestational age, birthweight and Apgar score.

Fit indices		Model 1	Model 2	Model 3
Absolute Index	Fit Function	0.03	0.03	0.03
	Chi-Square	11.00	9.64	10.63
	Pr > Chi-Square	0.20	0.29	0.22
Parsimony Index	RMSEA Estimate	0.03	0.02	0.03
Incremental Index	Bentler Comparative Fit Index	0.99	0.99	0.99
	Bentler-Bonett Normed Fit Index	0.95	0.96	0.95

Model 1: Gestational age; Model 2: Birthweight; Model 3: Apgar score.

## Data Availability

Data used in this manuscript is not publicly available, but access may be requested. Contact W.C.W.R.Z., M.Y.L. or J.K.W. for information.
